# CAR-macrophage versus CAR-T for solid tumors: The race between a rising star and a superstar

**DOI:** 10.17305/bb.2023.9675

**Published:** 2024-06-01

**Authors:** Kun Chen, Min-ling Liu, Jian-cheng Wang, Shuo Fang

**Affiliations:** 1 School of Medicine, Sun Yat-sen University, Shenzhen, China; 2 Department of Oncology, The Seventh Affiliated Hospital Sun Yat-sen University, Shenzhen, China; 3 Scientific Research Center, The Seventh Affiliated Hospital Sun Yat-sen University, Shenzhen, China

**Keywords:** CAR-macrophage, chimeric antigen receptor T cell (CAR-T), chimeric antigen receptor (CAR), adoptive cell therapy (ACT), tumor immunotherapy, solid tumor, tumor microenvironment (TME)

## Abstract

Adoptive cell therapy (ACT) has been demonstrated to be one of the most promising cancer immunotherapy strategies due to its active antitumor capabilities in vivo. Engineering T cells to overexpress chimeric antigen receptors (CARs), for example, has shown potent efficacy in the therapy of some hematologic malignancies. However, the efficacy of chimeric antigen receptor T cell (CAR-T) therapy against solid tumors is still limited due to the immunosuppressive tumor microenvironment (TME) of solid tumors, difficulty in infiltrating tumor sites, lack of tumor-specific antigens, antigen escape, and severe side effects. In contrast, macrophages expressing CARs (CAR-macrophages) have emerged as another promising candidate in immunotherapy, particularly for solid tumors. Now at its nascent stage (with only one clinical trial progressing), CAR-macrophage still shows inspiring potential advantages over CAR-T in treating solid tumors, including more abundant antitumor mechanisms and better infiltration into tumors. In this review, we discuss the relationships and differences between CAR-T and CAR-macrophage therapies in terms of their CAR structures, antitumor mechanisms, challenges faced in treating solid tumors, and insights gleaned from clinical trials and practice for solid tumors. We especially highlight the potential advantages of CAR-macrophage therapy over CAR-T for solid tumors**.** Understanding these relationships and differences provides new insight into possible optimization strategies of both these two therapies in solid tumor treatment.

## Introduction

In recent years, adoptive cell therapies (ACTs), especially chimeric antigen receptor T cell (CAR-T) therapy, have emerged as promising approaches for cancer immunotherapy. CAR-T cells, designed to overexpress chimeric antigen receptor (CAR), can recognize tumor-associated antigens (TAAs) and trigger targeted antitumor responses via extracellular single-chain variable fragment (scFv), a hinge domain, a transmembrane domain, and cytoplasmic signaling domain(s) [1, 2]. CAR-T therapy has shown potent efficacy in the therapy of hematologic malignancies. To date, all seven United States Food and Drug Administration (FDA)-approved CAR-T therapies are compatible with hematologic malignancies, such as acute lymphoblastic leukemia (ALL), mantle cell lymphoma, diffuse large B cell lymphoma (DLBCL), follicular lymphoma, and multiple myeloma. They all substantially improve the prognoses of patients [3–9]. However, CAR-T therapy faces significant challenges. Firstly, immunosuppressive tumor microenvironments (TMEs), such as immunosuppressive cytokines and checkpoints, diminish the antitumor efficacy of CAR-T cells and lead to their exhaustion [10–12]. Second, CAR-T cells struggle with infiltrating tumor sites due to the decreased adhesion molecules on the vascular wall and the dense extracellular matrix (ECM) surrounding tumor cells [13]. Third, the lack of TAAs or tumor-specific antigens (TSAs) targets with both high specificity and reliable safety pose further challenges [14, 15]. Other issues, such as antigen escape and severe side effects, also limit CAR-T therapy for solid tumors. We delve deeper into these concerns in the sections titled “Challenges Faced in Treating Solid Tumors” and “Adverse Events of CAR-T and CAR-macrophage Therapies.”

Interestingly, engineered macrophages that target solid tumors are potential candidates for overcoming some of these barriers, since macrophages naturally account for most of the tumor-infiltrating immune cells in many cancers [16]. Although in cancer contexts, naturally termed tumor-associated macrophages (TAMs), often display anti-inflammatory phenotypes [17, 18], their phenotypic plasticity allows them to be re-engineered to display antitumor activities [19]. This reprogramming can be achieved through recombinant expression of cytokines like Interleukin 12 (IL-12), Interferon alpha (IFN-α), or Interleukin 21 (IL-21) [20–22], overexpressing secreted cytotoxic agents [23], or inhibiting immunosuppressive genes like SIRPα with CRISPR-Cas9 [24]. Among these, CAR-macrophage is likely to be the most promising engineered macrophage therapy for solid tumors due to their antigenic specificity, infiltrating persistence, and other advantages that we will discuss in the sections “Antitumor Mechanism” and “Challenges Faced in Treating Solid Tumors.”

In this review, we aim to elucidate the current understanding of CAR-T and CAR-macrophage therapies, particularly the potential advantages of CAR-macrophages may have over CAR-T. We will also explore optimization strategies for treating solid tumors, drawing insights from both therapeutic approaches.

## CAR structures

### CAR structure in CAR-T cells

**Figure 1.**
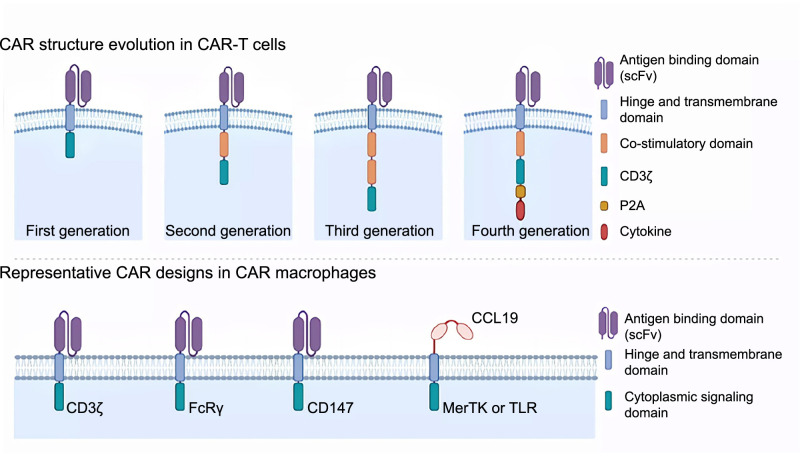
**CAR structures of CAR-T and CAR-macrophage.** CAR-T cells have undergone four generations of development (top panel), with each generation possessing distinct cytoplasmic domains. The first-generation CAR-T cells only express CD3^ζ^ as their cytoplasmic signaling domain. The second-generation CAR-T cells incorporate a costimulatory domain, such as CD28 or 4-1BB, to provide a second signal for T cell activation. The third-generation CAR-T cells have two costimulatory domains. The fourth-generation CAR-T cells, in addition to CD3^ζ^ and costimulatory domains, have a cytokine sequence downstream of CD3^ζ^, which enables them to produce proinflammatory cytokines (e.g., IL-7, IL-33, and IL-12) upon antigen recognition. A P2A peptide is added to cleave the cytokine from the antigen receptor. The CAR design employed in CAR-macrophages comprises an antigen binding domain, a hinge region, a transmembrane domain, and a cytoplasmic signaling domain, and exhibits great diversity in their cytoplasmic signaling domains (bottom panel). The signaling domains of CD3^ζ^ and FcRγ can mediate antigen-specific phagocytosis, while CAR-macrophages that utilize CD147 as a signaling domain can secrete matrix metalloproteinases (MMPs) to break down the dense extracellular matrix surrounding solid tumors. Additionally, in a recent study by Niu et al., CCL19, instead of single chain variable fragment (scFv), was utilized as the antigen-binding domain for CAR-macrophages to target an immunosuppressive cell population highly expressing CCR7. (This figure was created at BioRender.com.) CAR: Chimeric antigen receptor; CAR-T: Chimeric antigen receptor T cell; CAR-macrophage: Macrophages expressing CAR; CCR7: C-C motif chemokine receptor 7; FcRγ: Fc receptor gamma chain; IL-7: Interleukin 7; IL-33: Interleukin 33; IL-12: Interleukin 12.

The conventional CAR utilized in CAR-T cells comprises four domains: an antigen-binding domain, a hinge region, a transmembrane domain, and one or multiple cytoplasmic signaling domains (Figure 1).

The antigen-binding domain is typically composed of variable regions from both the heavy and light chains of a monoclonal antibody. These variable regions are conjoined together by a short linker to form an scFv. The scFv serves as a replacement for the T cell receptor (TCR) and can bind specifically to antigens, bypassing the need for a major histocompatibility complex (MHC). In some cases, the antigen-binding domain is derived from natural receptor-ligand pairs. For instance, D’Aloia et al. equipped second-generation CAR-T cells with the extracellular domain of FcγRIII (also known as CD16). This receptor is typically expressed on natural killer cells and facilitates antibody-dependent cell-mediated cytotoxicity (ADCC). When combined with monoclonal antibodies targeting tumor antigens, the extracellular FcγRIII domains of these CAR-T cells have demonstrated the ability to activate T cells, resulting in cancer cell death via ADCC [25].

The hinge region and the transmembrane domain, both usually derived from CD8, anchor the CAR in the T cell membrane and expose the antigen-binding domain on the cell surface. Interestingly, some studies suggest that the designs of the hinge region and transmembrane domain can also regulate CAR expression and signaling threshold of CAR-T cells [26].

The cytoplasmic signaling domain(s) transduce downstream signals to activate CAR-T cells, which eventually leads to tumor cell cytotoxicity [1, 2, 27]. First-generation CAR-T cells possess a single CD3^ζ^ domain, which provides the primary signal for T cell activation through its immunoreceptor tyrosine-based activation motif (ITAM). To enhance the antitumor activity and persistence of CAR-T cells, second-generation CAR-T cells typically contain an additional costimulatory domain (third-generation CAR-T cells contain two), along with CD3^ζ^, which is usually either CD28 or 4-1BB (CD137) [27–29]. Some researchers also introduced CD3ɛ into second-generation and promote the persistence of CAR-T cells [30]. Currently, the second-generation CAR stands as the most prevalent choice in both CAR-T research and clinical trials [2, 31]. Furthermore, advancements in CAR design have given rise to third-generation, fourth-generation, and other innovative structures, such as Split CARs, iCARs, and SynNotch CARs. These designs aim to heighten antitumor efficacy and mitigate severe side effects [32–45]. Detailed discussions regarding how these novel CAR designs address previous limitations will be presented in subsequent sections, specifically the “Antitumor Mechanism” and “Challenges Encountered in the Treatment of Solid Tumors.”

### CAR structure in CAR-macrophages

The CAR structure used in CAR-macrophages encompasses an antigen-binding domain, a hinge region, a transmembrane domain, and a cytoplasmic signaling domain. There is considerable diversity in their cytoplasmic signaling domains, as depicted in Figure 1.

Similar to CAR-T cells, the antigen-binding domain in CAR-macrophages is conventionally assembled using the variable regions of monoclonal antibody heavy and light chains, which are fused by a linker. Nonetheless, some researchers have explored the feasibility of employing natural receptor-ligand pairs as the antigen-binding domain for CAR-macrophages. In a recent study by Niu et al., C-C motif chemokine ligand 19 (CCL19) was utilized as an antigen-binding domain for CAR-macrophages to target an immunosuppressive cell population highly expressing C-C motif chemokine receptor 7 (CCR7), the ligand of CCL19. These anti-CCR7 CAR-macrophages showed significant suppression of tumor progression in a subcutaneous 4T1 breast cancer model [46].

The antigen-binding domain, hinge region, and transmembrane domain of CAR-macrophages all show minimal distinction from those of the first-generation CAR-T cells [47, 48]. However, CAR-macrophages demonstrate more diversity in their cytoplasmic signaling domains.

The cytosolic domain of CD3^ζ^ bears significant homology with the natural Fc receptor gamma chain (FcRγ), which mediates opsonization [48]. Hence, not surprisingly, activation domains of CD3^ζ^ and FcRγ are the most frequently used cytoplasmic signaling domains in CAR-macrophages, both triggering effective antigen-specific phagocytosis.

However, researchers have also explored alternative signaling domains that are peculiar to CAR-macrophages, endowing CAR-macrophages with some exclusive antitumor mechanisms. One of the most representative examples is the activation domain from CD147. Instead of triggering phagocytosis, this signaling domain upregulates the expression and secretion of matrix metalloproteinases (MMPs). MMPs can degrade the dense ECM surrounding solid tumors, facilitating immune cell infiltration into the tumor site [49]. Other alternative signaling domains include tyrosine-protein kinase Mer (MerTK) [50], multiple EGF-like-domains protein 10 (Megf10) [50], and toll-like receptors [46], which induce phagocytosis or proinflammatory cytokines secretion. The diverse cytoplasmic signaling domains of CAR-macrophages enable them to perform some unique antitumor mechanisms, which we will discuss later.

## Antitumor mechanism

The antitumor mechanisms of CAR-T cells are akin to those of natural effector T cells, encompassing the perforin-granzyme pathway, death receptor pathway, as well as cytokine secretion. Upon CAR recognition of tumor antigens, CAR-T cells induce tumor cell cytotoxicity via the perforin-granzyme pathway (intrinsic pathway of apoptosis) or death receptor pathway (extrinsic pathway of apoptosis) [33]. Apoptotic tumor cells release new tumor antigens (epitope spreading), which are subsequently engulfed by antigen-presenting cells (APCs). APCs then present these new antigens to endogenous T cells [1]. CAR-T cells also interact with other immune cells and tumor stroma to mediate tumor killing by secreting cytokines [33]. Traditional CAR-T cells interact with TME mainly by secreting interferon gamma (IFN-γ), which limits the diversity and intensity of the interaction between CAR-T cells and TME. Recent research efforts have also optimized CAR-T cells to secrete other proinflammatory cytokines (e.g., IL-7, IL-33, and IL-12). These modified CAR-T cells are referred to as TRUCK T cells or fourth-generation CAR-T cells, featuring a transgenic “payload” [32–38].

**Figure 2.**
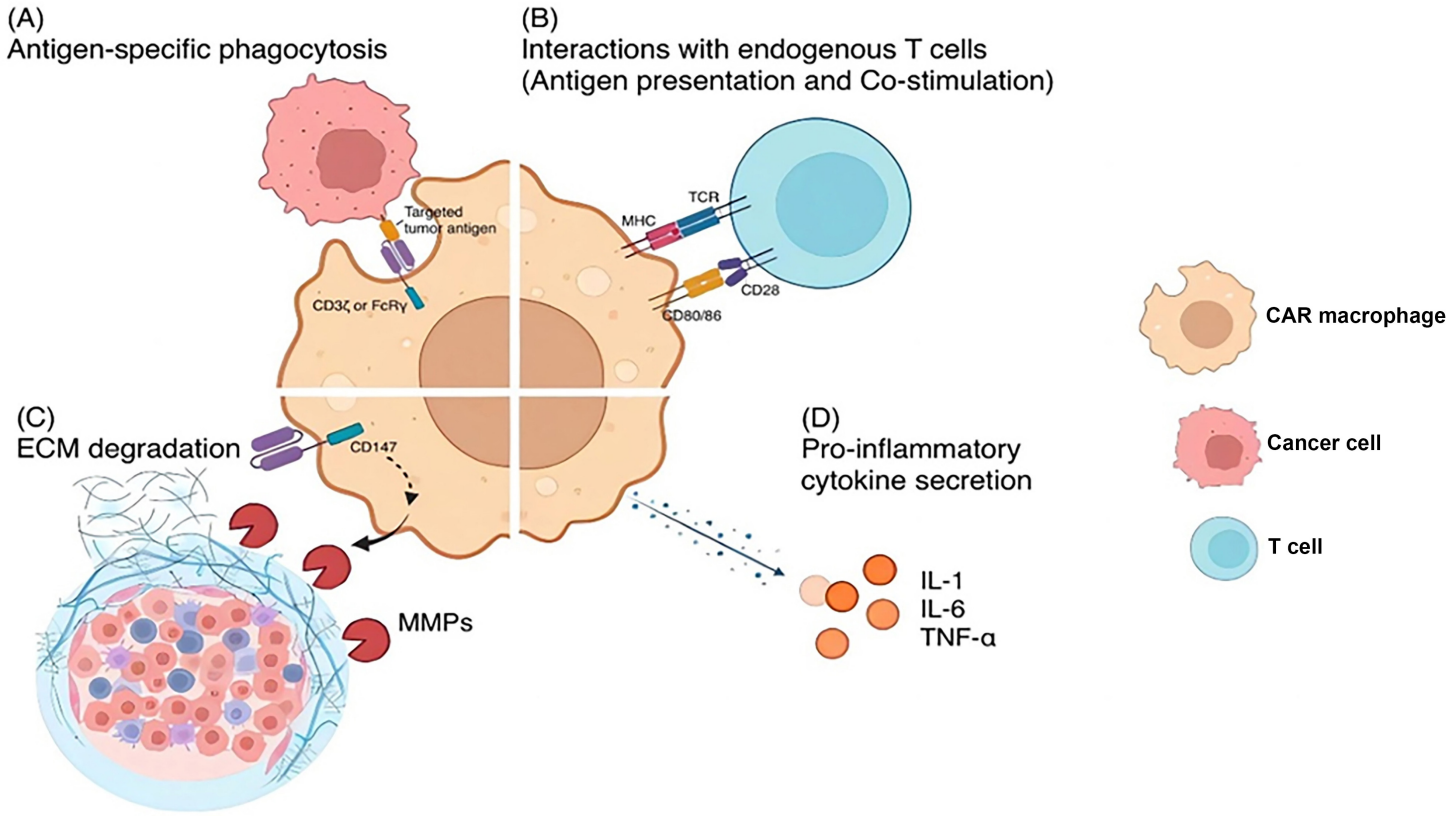
**Antitumor mechanisms of CAR-macrophage therapy.** The antitumor mechanisms of CAR-macrophages can be characterized by four main aspects. (A) CAR-macrophages directly kill tumor cells via antigen-specific phagocytosis mediated by the chimeric antigen receptor; (B) CAR-macrophages exhibit profound interactions with endogenous T cells. On the one hand, they present phagocytosed tumor antigens to T cells, and on the other hand, CAR-macrophages may facilitate the activation and persistence of endogenous T cells by costimulation; (C) When employing CD147 as the signaling domain, CAR-macrophages can secrete matrix metalloproteinases (MMPs) that degrade the dense extracellular matrix surrounding solid tumors, thereby facilitating the infiltration of other immune cells; (D) CAR-macrophages exhibit an M1-like phenotype, allowing them to secrete proinflammatory cytokines (e.g., IL-1, IL-6, and TNF-α) to educate the tumor microenvironment. (This figure was created at BioRender.com.) CAR-macrophage: Macrophages expressing CAR; IL-1: Interleukin 1; IL-6: Interleukin 6; TNF-α Tumour necrosis factor alpha; CAR: Chimeric antigen receptor; ECM: Extracellular matrix.

CAR-macrophages, however, trigger more variable antitumor mechanisms compared to CAR-T cells (Figure 2). By specifically directing macrophages toward tumor cells, CAR-macrophages can exhibit antigen-specific phagocytosis and cytotoxicity, mediating the direct killing of tumor cells. The cytotoxicity of macrophages is mainly mediated by the secretion of TNF-α. These mechanisms are similar to those observed in endogenous macrophages.

However, the distinct advantages of CAR-macrophage therapy over CAR-T stem from its unique and indirect antitumor mechanisms, particularly in addressing solid tumors. CAR-macrophages engage deeply with other immune cells. In vivo, for instance, CAR-macrophages recruit T cells to the tumor site through chemokine secretion. Studies have shown that compared to untransduced macrophages, CAR-macrophages promote superior chemotaxis of both resting and activated T cells toward tumor sites in humanized mice [47]. Once the T cells arrive, CAR-macrophages also maintain their APC capabilities, continuing to present tumor antigens to T cells. In a humanized immune system mouse model, CAR-macrophages were found to highly express MHC-II at the tumor sites, while the natural macrophages showed a low expression of MHC-II [47].

More importantly, CAR-macrophages manifest an M1 phenotype due to the adenovirus-based transduction process [47]. This enables them to educate the TME through the expression of proinflammatory cytokines and costimulatory molecules, amplifying the antitumor actions of both endogenous macrophages and other immune cells. Research indicates that CAR-macrophages can induce a phenotypic shift in M2 macrophages via interferon signaling, iNOS signaling, and the Th1 pathway [47, 51]. In addition, CAR-macrophages express significantly higher levels of CD80/CD86 than natural macrophages, thus bolstering not only the activity but also the infiltration and persistence of antitumor T cell response [47].

Finally, CAR-macrophages are capable of revolutionarily targeting the ECM of the tumor site through the release of MMPs. It is well established that TAMs, exhibiting M2 phenotypes, are prompted by certain cancer cells to construct a dense ECM around the tumor [52–54]. As highlighted above, CAR-macrophages exhibit M1 phenotypes, which prevent the formation of dense ECM at the tumor site. Furthermore, CD147+ CAR-macrophage upregulates the secretion of MMPs during antigen recognition, thereby degrading the ECM of the tumor [49]. This novel mechanism provides a promising strategy for cancer immunotherapy: tumor ECM density reduction promotes infiltration of other immune cells, such as dendritic cells, T cells, and natural killer cells.

In conclusion, the primary advantage of CAR-macrophage therapy over CAR-T for the treatment of solid tumors lies in the diverse antitumor mechanisms, including the extensive interactions with other immune cells (by secreting various proinflammatory cytokines or acting as APC), the ability to potently reactivate TME, the capability to promote the persistence of T cell infiltration, and, finally, a novel strategy to target the tumor ECM. With these CAR-macrophage-exclusive antitumor mechanisms, CAR-macrophage therapy even showed potent efficacy in treating cold tumors, such as breast cancer, as it has been seen in several pre-clinical studies [47, 49, 55, 56].

## Challenges faced in treating solid tumors

Although CAR-T therapy has demonstrated remarkable efficacy in treating B cell lymphoma, it faces considerable challenges when treating solid tumors, some of which are also encountered by CAR-macrophage therapy.

### Scarcity of suitable tumor antigens

A significant obstacle common to both CAR-T and CAR-macrophage therapies pertains to the scarcity of suitable TSAs or TAAs [50–52]. The identification of suitable TSAs and TAAs is hampered by significant obstacles, specifically, on-target off-tumor toxicity, the antigen escape phenomenon, and intratumor antigen heterogeneity [13, 57–59]. These barriers are hereby further discussed in detail.

Namely, the targeted solid tumor antigen should have both high specificity and reliable safety. Otherwise, CAR-T therapy may cause a kind of toxicity known as on-target off-tumor, which means the CAR-T cells may attack normal tissues expressing the targeted antigen [57, 60]. Various approaches have been devised by researchers to address the issue of on-target off-tumor toxicity [61]. These include Split CARs, which endow two distinct TAAs with the capacity to trigger the first or second signal for CAR-T cell activation [41, 42, 45]; iCARs, which enable a non-tumor antigen to facilitate a coinhibitory signal in CAR-T cells [40]; and SynNotch CARs, which make CAR expression dependent on the interaction between a different TAA and another engineered synNotch receptor [43, 44]. There are still other strategies to limit on-target off-tumor toxicity that have been reviewed elsewhere [57].

Moreover, the phenomenon of antigen escape further complicates the quest for suitable tumor antigens. Once CAR-T cells begin antigen-specific eradication, tumor cells may downregulate the expression of the targeted antigen, resulting in antigen escape. This phenomenon substantially undermines the efficacy of the TAA or TSA and holds the potential to endanger tumor recurrence [58, 62]. To overcome this challenge, researchers have developed CAR-T cells that target two or more antigens (bispecific or multispecific CAR-T), which have shown promising results in some pre-clinical studies [62, 63].

Finally, intratumor antigen heterogeneity also complicates the search for suitable tumor antigens. Intratumor antigen heterogeneity refers to the variation in surface antigen expressions among distinct cancer cells within the same tumor. If the antigen targeted by CAR-T cells is not expressed on all cancer cells within a tumor, intratumor antigen heterogeneity can potentially lead to tumor recurrence [59].

### Inadequate and nonpersistent CAR-T cell infiltration

Beyond the scarcity of appropriate TSAs and TAAs, another significant barrier to solid tumor-targeting CAR-T is the inadequate and non-persistent infiltration of CAR-T cells into the tumor site. The dense ECM often obstructs CAR-T cell infiltration. Moreover, the immunosuppressive TME, characterized by immunosuppressive cytokines (e.g., IL-10 and TGF-β) and checkpoints (e.g., PD-1 and CTLA-4), can lead to an exhausted CAR-T cell phenotype when targeting solid tumors [10, 11, 13]. Researchers explored multiple strategies to counteract the immunosuppressive microenvironment [61, 64], including CAR-T cell endowment with the ability to secret chemokines and proinflammatory cytokines [32], pairing one type of CAR-T with another cancer-associated fibroblast (CAF)-targeting CAR-T [65, 66], and equipping CAR-T cells with additional costimulatory molecules like OX40 [67].

### Potential advantages and challenges of CAR-macrophage therapy

The limitations of CAR-macrophage therapy for solid tumors have been less studied compared to CAR-T therapy, with only one clinical trial currently underway. However, CAR-macrophage shows promise for solid tumor treatment, mainly due to the natural abundance of macrophages at the tumor site. In fact, macrophages have the greatest infiltration among various immune cells at the tumor site [16]. While many naturally infiltrating macrophages tend to display anti-inflammatory phenotypes, often manipulated by tumor cells expressing suppressive molecules like CD47 (“don’t eat me”) [68–73], CAR-macrophages seem to have the ability to overcome these problems. As previously discussed, the adenovirus-transduced CAR-macrophages predominantly adopt M1 phenotypes [47]. While the CD47 don’t eat me signal can be blocked by depleting the downstream SIRPα gene [24], it is plausible to assume that other suppressive pathways may also be blocked by similar methods.

Consequently, the ability for abundant and sustained infiltration into solid tumors is another edge that CAR-macrophage therapy holds over CAR-T in this context. However, challenges like the limited availability of suitable TSAs or TAAs and the antigen escape issue are expected to be common to both CAR-macrophage and CAR-T therapies.

## Insights from clinical trials and practice for solid tumors

### CAR-T and CAR-macrophage clinical trials for solid tumors: A brief overview

To date, there are over 200 registered clinical trials globally investigating CAR-T cell therapies for solid tumors, with a majority being Phase 1 trials aimed at assessing the safety and efficacy of different CAR-T products. Glioma, pancreatic, lung, breast, and prostate cancers are the most frequently selected tumor types for evaluation. Among the CAR-T platforms, the second-generation CAR-T cells are the most frequently employed, but third- and fourth-generation CAR-T cells also see significant utilization in treating solid tumors [31]. The most frequently targeted antigens are human epidermal growth factor receptor 2 (HER2), mesothelin, and claudin18.2, with 4-1BB being the most frequently utilized costimulatory domain. Notably, most of the trials targeting solid tumors focus on single-target CAR-T cells, with only nine studies evaluating the efficacy of targeting more than two antigens concurrently [31].

Through a comprehensive search of the PubMed database utilizing the keywords “CAR-T” and “solid tumor,” we identified 18 CAR-T clinical trials for solid tumors that reported their preliminary or final outcomes. Our search reveals that all these trials with reported results are designed as Phase I clinical trials, treating various solid malignancies, including gastrointestinal, prostate, breast, lung, and pancreatic cancers. While preliminary results suggest promising antitumor efficacy of CAR-T products, the occurrence of grade 1–3 cytokine release syndrome (CRS) is a frequent adverse event among the trial participants [38, 74–81]. Additionally, there has been one death case due to grade 4 CRS in a trial evaluating prostate-specific membrane antigen-targeting CAR-T for prostate cancer [77].

Furthermore, ongoing CAR-T clinical trials for solid tumors that have not yet reported their results are also worth noting. A search on the ClinicalTrials.gov website retrieved 129 such trials. Among these, 92 were phase 1, and 37 were phase 1/2 trials. Within this spectrum of ongoing clinical trials, several employ innovative CAR-T cell designs. For instance, in a phase 1/2 trial led by Feng et al. (NCT05693844), which plans to recruit 30 participants, researchers aim to evaluate the safety and efficacy of CAR-T cells targeting mesothelin and expressing CD40 ligand in the treatment of advanced/metastatic solid tumors. These CAR-T cells are anticipated to reduce CAR-T cell exhaustion and enhance the antitumor effects of endogenous APCs, including dendritic cells and macrophages. Moreover, in another phase 1/2 trial (NCT05681650) that plans to enroll 30 participants, investigators utilize a novel hypoxia-stimulated CAR expression system to target HER2-positive solid tumors. This system enables CAR-T cells to effectively expand and survive in the hypoxic TME. While it has shown promise in preclinical studies, the novel approach of improving CAR-T cell adaptability to the hypoxic microenvironment has not yet been clinically validated. The trial will assess if this approach indeed enhances CAR-T cells’ ability to treat solid tumors in humans.

In comparison to CAR-T, the clinical experience and data available for CAR-macrophage therapy are limited. As of now, only one clinical trial with CAR-macrophage therapy has been conducted, recruiting 18 subjects (NCT04660929). The preliminary results of this trial were reported in November 2021 and demonstrated that CAR-macrophages have the capacity to secrete proinflammatory cytokines, reprogram the TME, recruit innate immune cells and naïve T cells, and enhance the infiltration and persistence of CD8+ T cells [82].

### Adverse events of CAR-T and CAR-macrophage therapies

The use of CAR-T therapy has consistently shown a high incidence of adverse events in clinical trials for both hematologic malignancies and solid tumors, as documented in prior studies [57, 61, 83, 84]. Amongst these, CRS and immune effector cell-associated neurotoxicity syndrome (ICANS) are the most frequently observed toxicities linked to CAR-T therapy. CRS presents as a clinical syndrome of fevers, hypotension, hypoxia, and neurologic changes and is caused by the secretion of proinflammatory cytokines, including IL-6, IL-8, monocyte chemoattractant protein-1 (MCP-1), and macrophage inflammatory protein-1α (MIP-1α) [1, 85]. In a phase 1 clinical trial assessing the safety and efficacy of claudin18.2-specific CAR-T cells for gastrointestinal cancers (NCT03874897), 35 out of 37 patients (94.6%) experienced grade 1 or 2 CRS, with no recorded instances of grade 3 or higher CRS [78].

ICANS may arise due to increased cytokine levels in the cerebrospinal fluid and disruptions to the blood–brain barrier, resulting in neurological symptoms, such as aphasia, tremors, seizures, headache, and in severe cases, life-threatening cerebral edema [61, 86]. Whilst several measures (such as tocilizumab, an IL-6R inhibitor) received the FDA approval to manage CAR-T-induced adverse events, both CRS and neurotoxicity can still pose life-threatening risks to patients undergoing CAR-T therapy [1].

Although frequently occurring in CAR-T therapies for solid tumors, CRS and ICANS seem to be comparatively less severe than in hematologic malignancies. The primary and most severe adverse event in CAR-T therapies for solid tumors is on-target off-tumor toxicity, whereby CAR-T cells can attack normal tissues expressing the targeted antigen [57]. Depending on the antigen targeted, liver gastrointestinal, and life-threatening pulmonary toxicity have been reported in different solid tumor CAR-T clinical trials, including those targeting carboxy-anhydrase-IX, carcinoembryonic antigen (CEA), HER2, or claudin18.2. [78, 87–89]. In a phase 1 clinical trial assessing the safety and efficacy of carcinoembryonic antigen (CEACAM5)-specific CAR-T cells in the treatment of digestive tract carcinoma (NCT01212887), 4 out of 14 patients (28.6%) experienced pulmonary toxicities identified as suspected unexpected serious adverse reactions (SUSARs), presenting with respiratory distress [89]. The discovery of highly specific tumor antigens is essential to minimize the risk of on-target off-tumor toxicity. Moreover, numerous engineering strategies have been developed to mitigate on-target off-tumor toxicity, as previously reviewed.

In contrast, the potential side effects of CAR-macrophage therapy remain uncharted, with only one ongoing clinical trial to date. This trial, led by Klichinsky et al. (NCT04660929), revealed no significant organ toxicities in the two treated subjects. However, one subject experienced grade 2 CRS on day 3 which resolved on the same day [82]. Given the innate cytokine-secreting ability of macrophages, it is anticipated that CAR-macrophage therapy might induce CRS, an expectation set early in CAR-macrophage development. Drawing from experiences with CAR-T, it would be wise to explore possible solutions to CRS induced by CAR-macrophages. Lastly, more clinical trials are necessary to further evaluate the potential side effects of CAR-macrophage therapy.

## Optimization strategies

Upon reviewing the similarities and differences between these two ACTs (Table 1), the aforementioned comparative review can potentially inspire novel optimization strategies for both CAR-T and CAR-macrophage therapy. In this discussion, our primary focus is on the strategies that these therapies may mutually benefit from, as other promising optimization strategies have been reviewed elsewhere [48, 90–92].

**Table 1:** Comparison of CAR-T and CAR-macrophage for solid tumors

**Categories of comparison**	**CAR-T**	**CAR-macrophage**
Cytoplasmic signaling domain	Costimulatory domain(s) and CD3^ζ^; CD3ɛ may promote persistence	Usually CD3^ζ^ or FcRγ; MerTK, Megf10, TLRs, and CD147 are alternative
Major antitumor mechanisms	Perforin-granzyme or death receptor-mediated cytotoxicity	1) Antigen-specific phagocytosis; 2) TME “education”; 3) ECM degradation
Chemokine secretion	No	Yes
Cytokine secretion	Only IFN-γ; but TRUCK T cells can secret other proinflammatory cytokines	Proinflammatory cytokines including IL-1, IL-6, and TNF-α
Costimulation	–	Yes
Antigen presentation	–	Yes
ECM degradation	Usually no; but CAF-targeted CAR-T can shrink the ECM	Yes, with CD147 as the signaling domain
Infiltration into solid tumor	Poor	Abundant
Appropriate TSAs or TAAs	Scarce	Scarce
Antigen escape	Common	Predicted to be common
Major side effects	CRS, neurotoxicity, and OTOT	CRS observed; clinical data are insufficient
Clinical trials for solid tumor	Over 200	Only 1

TLRs: Toll-like receptors; TME: Tumor microenvironment; ECM: Extracellular matrix; TRUCK: T cells redirected for universal cytokine-mediated killing; CAF: Cancer-associated fibroblast; TSAs: Tumor-specific antigens; TAAs: Tumor-associated antigens; CRS: Cytokine release syndrome; OTOT: On-target off-tumor toxicity; CAR-T: Chimeric antigen receptor T cell; CAR-macrophage: Macrophages expressing CAR; FcRγ: Fc receptor gamma chain; MerTK: Tyrosine-protein kinase Mer; Megf10: Multiple EGF-like-domains protein 10; IFN-γ: Interferon gamma; IL: Interleukin; TNF-α: Tumour necrosis factor alpha.

### Solid tumor CAR-T optimization: Inspired by CAR-macrophage

#### Reprogramming tumor-associated macrophages during CAR-T therapy

It was demonstrated that TAMs are predominant immune cells infiltrating tumor sites [16]. Typically, they undermine T cell-mediated antitumor immunity by releasing cytokines such as IL-10 and TGF-β and expressing coinhibitory molecules like PD-L1, PD-L2, B7-H4, and VISTA [93]. TAMs can also promote Tregs infiltration by secreting CCL20 and CCL22, further suppressing the antitumor activity of T-cells [18, 94]. Moreover, some in vitro studies showed that TAMs can induce CD4+CD25- tumor-infiltrating T cells to Tregs [95].

Explorations into CAR-macrophage and other engineered macrophage therapies have delved into the phenotypic plasticity of macrophages, assessing the potential to convert immunosuppressive TAMs into a proinflammatory M1-like phenotype. These M1-like macrophages are characterized by their phagocytic and antigen-presenting capabilities, and their ability to modify the TME by discharging proinflammatory cytokines like TNF-α, IL-1, IL-6, and IL-12 [64]. The combination of CAR-T cells and reprogrammed macrophages has been explored as a potential approach to enhance antitumor activity. For example, Xie et al. engineered CAR-T cells to secret anti-CD47 single-domain antibody fragments (CD47 is usually expressed on tumor cells and conveys a “don’t eat me” signal to TAMs). This modification substantially enhanced the phagocytic activity of macrophages and other myeloid cells, and successfully increased the infiltration of M1-like macrophages within the tumor [96]. Consequently, in contrast to conventional CAR-T, this antibody-secreting CAR-T manifested superior antitumor activity and extended survival time [96]. Other researchers engineered CAR-T cells to constitutively express CD40L, which enhances IL-12 secretion by macrophages in a lymphoma model [97, 98]. It’s plausible to infer that similar modifications could pivot TAMs toward a proinflammatory phenotype in solid tumor-targeting CAR-T therapies. In another preclinical study, Spear et al. [99] demonstrated that the CAR-T-cell-driven secretion of GM-CSF and IFN-γ enhances the capabilities of IL-12 secretion and antigen-presenting of TAMs, transforming the TME from immunosuppressive to immunostimulatory state in an ovarian tumor model.

A more audacious strategy in CAR-T therapy is to directly target TAMs in CAR-T therapy: enabling CAR-T to eliminate the immunosuppressive M2-like TAMs within the tumor site. Several TAM-related antigens (e.g., folate receptor β [FRβ] and B7-H4) have been found to achieve this strategy by some preclinical studies [64, 100, 101]. Regardless, CAR-T therapies that target these antigens have shown either minimal improvement of antitumor efficacy or severe toxicities [100, 101], indicating that directly depleting TAMs by CAR-T cells may not be a safe and optimal strategy.

#### Targeting the dense extracellular matrix around solid tumor

Another avenue for refining CAR-T cell therapy is targeting the ECM surrounding solid tumors. As previously discussed, dense ECM presents a significant physical barrier to CAR-T cell infiltration and antitumor activity, while CAR-macrophages have the ability to break down tumor stroma [49]. Hence, some studies focused on developing CAR-T cells that target CAFs, the major producers of the ECM around tumor cells [65]. In a study by Kakarla et al., CAR-T cell therapy against a fibroblast activation protein-α, a biomarker of CAFs, successfully shrank the ECM in the A549 lung cancer mouse model. When these CAR-T cells were coupled with another group of CAR-T cells that targeted the EphA2 antigen on the A549 cancer cells, significantly improved antitumor activity and survival rates in the mice were observed compared to the treatment with EphA2-specific CAR-T cells alone [102].

### CAR-macrophage optimization

#### CAR-macrophage combination therapies

Given that T cells, rather than macrophages, are the primary immune cells in cancer immunity, one could infer that the efficacy of CAR-macrophage monotherapy against solid tumors may be insufficient, despite its diverse antitumor mechanisms and abundant infiltration into tumor sites. Additionally, previous studies indicated that the antitumor effectiveness of CAR-macrophage therapy might be augmented through various combination therapies [48, 55, 103].

For instance, chemotherapy and radiotherapy have been shown to promote macrophage immunity by stimulating antigen presentation, inducing the expression of costimulatory molecules, and triggering the secretion of proinflammatory cytokines and chemokines [103–107]. Nevertheless, it is worth noting that current research findings suggest that the impact of radiotherapy on macrophage immunity may exhibit a dual nature. Although radiotherapy may induce the expression of chemokines, cytokines, and receptors associated with macrophage recruitment, these macrophages tend to acquire immunosuppressive phenotypes during the later phases of tumor immunity [108]. Crittenden et al. observed that tumor-infiltrating macrophages upregulated CCL2 and CCL7, both of which are linked to the recruitment of monocytes and macrophages. Unfortunately, these newly recruited macrophages demonstrated a propensity to polarize into immunosuppressive phenotypes due to a transcriptional shift mediated by the regulation of NFκB p50, which was induced by dying cancer cells [109].

Moreover, monoclonal antibody therapies targeting tumor antigens like HER2 can effectively activate macrophages to phagocytose opsonized tumor cells [48, 110]. In addition, antibodies that block phagocytosis-inhibiting signals, such as the “don’t eat me” (CD47) signal, can augment the phagocytosis of macrophages and other myeloid cells [96, 111, 112]. In a recent investigation by Upton et al., the synergistic application of anti-CD47 (magrolimab) and anti-HER2 (trastuzumab) antibodies displayed remarkable potential in eradicating HER2+ breast cancer cells, primarily due to the augmentation of ADCP by macrophages. This effect was observed even when the cancer cells displayed tolerance to trastuzumab-induced ADCC mediated by natural killer cells [110].

#### CAR-macrophage optimization inspired by CAR-T: Enhancing the activity of tumor-infiltrating T cells

T cells play a pivotal role in cancer immunity. The success of CAR-T therapy underscores how optimizing endogenous T cells can bolster antitumor immunity. It stands to reason that the concurrent reprogramming of endogenous tumor-infiltrating T cells may amplify the antitumor efficacy of CAR-macrophage therapy.

For instance, Pierini et al. combined CAR-macrophages with anti-PD-1 therapy, which inhibits the coinhibitory molecule PD-1 on T cells. Their findings revealed an increased survival rate in a colon cancer model, compared to CAR-macrophage monotherapy [55]. In another preclinical study, Gardell et al. engineered macrophages to secret a bispecific T cell engager that targets the mutated epidermal growth factor variant III (EGFRvIII) found in certain glioblastoma tumors. This strategy enhanced T cell activation, proliferation, and targeting of antigen-specific tumor cells by bridging the recognition between TCR and the tumor antigen [20, 113]. In theory, the overexpression of costimulatory molecules, such as CD40 or CD80/86 on CAR-macrophages could also enhance tumor-infiltrating T cells. However, as far as we know, no study has yet tested this, so additional studies are needed to ascertain this assumption.

## Future directions

Despite the various optimization strategies discussed so far, both CAR-T and CAR-macrophage therapies are still confronted with certain limitations, such as the scarcity of potent and secure TSAs or TAAs, as well as treatment-related adverse events, especially on-target off-tumor (OTOT) toxicity. To address these hurdles effectively and securely within the context of solid tumor cell therapy, further exploration is necessary. Comparative analysis of tumor and normal RNA through next-generation sequencing techniques, alongside employing immunopeptidomic methods and T cell-based detection, presents promising avenues for the identification of novel targeting antigens in solid tumors [114–116]. Through these emerging approaches, it is possible to identify tumor antigens known as neoantigens, which, compared to traditional TAAs, offer advantages, such as high specificity, improved security, and better personalization [115, 117]. Particularly for CAR-macrophage therapy, pinpointing new targeting antigens is a fundamental and potent approach to mitigate OTOT toxicity. Novel immunotherapeutic agents targeting tumor-site microenvironments are also revolutionizing cancer therapy.

Additionally, expanded clinical trials centered on solid tumors are necessary, aiming to provide valuable insights and a deeper understanding of the therapeutic potential and potential risks associated with both CAR-T and CAR-macrophage therapies.

## Conclusion

In this review, we compared the current state of research on CAR-T therapy and CAR-macrophage therapy for the treatment of solid tumors. While CAR-T therapy has been extensively studied both preclinically and clinically, CAR-macrophage therapy remains in its nascent stage. These two ACTs differ significantly in many crucial features, including but not limited to their CAR structures, antitumor mechanisms, challenges they encounter in treating solid tumors, as well as adverse events. By reviewing these differences, we conclude that CAR-macrophage therapy appears to have two major advantages over CAR-T therapy when targeting solid tumors: a broader range of antitumor mechanisms and superior infiltration into tumor sites. Yet, CAR-macrophage therapy is still expected to share some of the hurdles encountered by CAR-T therapy, such as the scarcity of appropriate TSAs or TAAs and the antigen escape phenomenon. Employing innovative approaches for the identification of novel tumor antigens holds the potential to surmount these common obstacles shared by both CAR-T and CAR-macrophage therapies. As we monitor the continued development of these two approaches to immunotherapy, we remain optimistic that their developments will reciprocally inform optimization strategies as they compete in the race to achieve effective and safe solid tumor immunotherapy.
